# Causal conditions for loneliness: a set-theoretic analysis on an adult sample in the UK

**DOI:** 10.1007/s11135-017-0482-y

**Published:** 2017-02-13

**Authors:** Keming Yang

**Affiliations:** School of Applied Social Sciences, Durham University, Durham, DH1 3HN UK

**Keywords:** Loneliness, Ageing, Causality, Qualitative comparative analysis (QCA), Set-theoretic methods

## Abstract

While age has been identified as a risk factor for loneliness, whether it is a necessary or sufficient condition for loneliness has never been examined. This is the first study that applies fuzzy-set QCA, a special type of set-theoretic method, to discover the necessary and sufficient causal conditions for loneliness, respectively, among adults in the UK, analysing the data collected from the UK sample of Round 6 of the European Social Survey (ESS, 2012, n = 2163). It firstly examines the configurations of five conditions: being female, old age, not living with spouse/partner, bad health, and not being frequently social with others. Gender was found neither a necessary nor a sufficient condition for loneliness, and old age was close to being a necessary condition and became necessary when united with any of the other conditions; the configuration of not living with spouse/partner and not healthy and not frequently social with others is a sufficient condition. Robustness of results was tested with two different conditions (a limiting illness and a confidante), and a separate analysis on the absence of loneliness was conducted. The effect of the unbalanced distribution of cases across different values of the outcome was highlighted as a source of uncertainty, and the results on the absence of loneliness are different from those on its presence.

## Introduction

Loneliness is an unpleasant psychological reaction to the absence of desired social relations (Anderson 1998; De Jong Gierveld 1987; Perlman and Peplau 1981; Townsend 1968; Victor et al. 2000; Weiss 1973), and medical researchers have established its detrimental effects on both physical as well as mental health. For example, the mortality differential between lonely and non-lonely individuals is as high as 50% (Holt-Lunstad and Smith 2012; Luo et al. 2012), the lonely have much higher rates of dementia and depression (Cacioppo et al. 2010; Prieto-Flores et al. 2011), the rate of GP consultation among the lonely is more than three times that of the non-lonely, and their emergency hospital admission rate is 30% higher than the non-lonely (Ellaway et al. 1999; Molloy et al. 2010). With as many as 800,000 people ‘chronically lonely’ in England, the health secretary Jeremy Hunt referred to loneliness as a source of ‘national shame’ in his speech to the National Children and Adults Services (NCAS) conference on October 18th 2013. As a result, the study of loneliness has become a burgeoning interdisciplinary field in the social sciences of ageing and public health.

Given these undesirable effects of loneliness, to find out what makes people feel lonely in the first place is naturally the next important question. Social gerontologists have made admirable progress in estimating the prevalence of loneliness and identifying risk factors for loneliness across different groups (Anderson 1998; Hawkley and Cacioppo 2010; Victor et al. 2000, to name just a few). Of existing studies on loneliness, most employ generalized linear regression models, although an increasing number of researchers in the area have implemented more complex models to account for the nature of the link between loneliness and health (for example, Luo et al. 2012). Some prominent social scientists (Freedman 1991; Hedström and Swedberg 1998) have pointed out serious limitations of such a statistical approach. Among the criticisms of linear statistical methods, the set-theoretic method in general and qualitative comparative analysis (QCA) in particular have developed into a challenging alternative to the statistical approach (Ragin 2000, 2008b). Whilst this relatively new approach is not a panacea—as explained later in this paper, it has limitations of its own—it is able to offer fresh insights into the causal conditions of the interested outcome. The key motivation behind this study is that the employment of set-theoretic methods will help us answer some important research questions that other studies on loneliness using statistical methods cannot, for example, which among the combinations of attributes such as gender, age, living with a spouse and self-reported health is a sufficient condition for feeling lonely, so that the new insights thus obtained will help tackle loneliness more effectively.

## How could QCA help research on loneliness?

As it is very likely that this is the first study that applies QCA for studying loneliness, a succinct introduction to how this method could benefit the research on loneliness seems desirable. The method has been successfully used in different areas, such as business administration (Chung 2001), public health (Longest and Thoits 2012), social policy (Blackman 2013) and sociology (Crowley 2013). The reader could learn more details about the method by reading the texts in References.

According to Ragin (1989, 2000, 2008b), the most enthusiastic advocate and developer of this method, QCA is a set-theoretic method because the cases under study are treated as members of a set, such as ‘older people’ or ‘widowers.’ Set membership can be either crisp—either in or out, represented by 1 or 0, respectively (*cs*QCA)—or fuzzy, i.e., the degree to which a case belongs to a set, usually represented by a decimal number (*fs*QCA). Set membership is not computed but determined by the analyst, and the process of assigning a numeric value to set membership is called ‘calibration.’ An example of crisp-set is ‘living alone,’ and the calibration of ‘older people’ as a fuzzy-set will be explained later. The simultaneous set memberships of the cases constitute ‘configurations,’ such as ‘being older’ and ‘widowed,’ which are taken as potential causal conditions for an interested outcome, such as ‘frequently lonely.’ A ‘truth table’ is constructed with all possible configurations put together with the corresponding percentages of cases that represent the outcome, which will become the target of all subsequent analyses. The objective of the analyst is to discover which of these configurations could be identified as either necessary or sufficient causal conditions for the outcome by conducting logical analyses of the truth table.

In contrast to generalized linear regression models, QCA has at least two major advantages. First, linear statistical models focus on the net (or unique) effect of each independent variable, such as age, gender, marital status, etc., on the dependent variable, such as loneliness score, based on statistics of controlled associations. In addition, few statistical models in the literature of loneliness include interaction-terms, and even if they do, an interaction-term is usually consisted of only two variables. As explained above, QCA analyses configurations which are essentially interactions of multiple (three or more) conditions (or variables). If statistical models take causation as ‘robust dependence’ (Goldthorpe 2001), then QCA studies ‘conjunctural causation’ (Schneider and Wagemann 2012: 6), that is, set-relationships between the configurations of conditions and the outcome. Some statistical models do analyse interactions, such as log-linear models and the related configurational frequency analysis (von Eye 2002), but their purpose is to find the model that best fits the data, not to discover which configurations are necessary or sufficient for a certain outcome. As illustrated below, QCA allows researchers to discover which among the possible configurations of multiple conditions (gender, age, etc.) could be identified as necessary or sufficient for the outcome set of ‘frequently feeling lonely.’

Another major advantage of QCA is its capacity to reveal asymmetrical causal relationship, although Stanley Lieberson (1987) already called for serious attention to this issue before the wide use of QCA. It is therefore necessary and useful to study the two opposite outcomes separately rather than to treat them as two values of the same variable. For the study of loneliness, this means that the *presence* of certain conditions for *feeling* lonely may not be directly inferred from the *absence* of these conditions for *not feeling* lonely. For example, being female, widowed and living alone may be a causal condition for feeling lonely, but that does not necessarily mean that males who are not widowed and living with someone would not feel lonely. Logically speaking, this may appear obvious, but symmetric reasoning is often implied in studies based on statistical associations.

Once the reader is familiar with the basic ideas of QCA, it should not be difficult to see that QCA is not exactly one of the ‘qualitative research methods’ which usually refer to the collection and analysis of a large amount of non-numeric data (texts and images). As explained above, the data for QCA consist of an outcome and the selected conditions whose values are between 0 and 1. Results of QCA are logical solutions, supported with statistics of consistency and coverage (to be defined below). When the number of cases becomes so large that it is impossible for the analyst to study each case with rich qualitative information, or when such information is not available, all that the analyst can rely on are these solutions and statistics. In this sense, QCA is quantitative. QCA has been labelled as ‘qualitative’ because originally it grew out of the small-N comparative approach, such as Skocpol’s (1979) well-known study on a handful of states and social revolutions. For these attributes, QCA is seen as an alternative to both statistical methods and small-N qualitative studies, and Ragin (2008b) has urged users of QCA not to use it as a purely technical method but a methodological approach as well.

The dual aims of increasing analytical power by applying formal logical procedures on the one hand and retaining qualitative richness on the other raise the question whether QCA could be applied on large-N datasets. In contrast to the data collected in social surveys, which are usually of a very large size (in thousands) and designed to represent a definite population, the cases analysed in most studies using QCA are not only of a small size (less than 50 or even 30) but also not randomly selected—inference from a representative sample to its population has not appeared to be an issue for QCA. In principle, there is no logical or technical reason for QCA and set-theoretic methods to impose a limit on the number of cases. In fact, the number of cases must be big enough in order to avoid too many logical remainders, i.e., configurations with no cases (Schneider and Wagemann 2012: 317). Emmenegger et al. argued that ‘the linguistic form of survey data often lends itself to a direct translation into fuzzy sets’ (2014: 3). Greckhamer et al. made a similar point: ‘Hypothetico-deductive large-N QCA applications are not only possible but in our view present one of the most promising areas to extend the set theoretic approach’ (2013: 55). Some researchers have demonstrated that QCA could indeed be applied on data of large size (Cooper 2005; Ragin and Fiss 2008; Glaesser and Cooper 2010; Cooper and Glaesser 2011, 2015; Greckhamer et al. 2013). However, the use of QCA on large-N data will be different from QCA on small-N data, as will be demonstrated in this paper.

It is important to note that the development of QCA has not been free of controversies. Lucas and Szatrowski (2014) produced a recent and perhaps the most systematic critical evaluation of QCA (see also Goldthorpe 2000; Lieberson 2001, 2004). As Ragin and others do not accept these criticisms (see their reactions published in the same issue of Lucas and Szatrowski 2014), including the deterministic nature of QCA, the effect of calibration of conditions, etc., the debate will continue. The author of this paper does not pretend to be able to resolve the controversy but does hold the view that for at least two reasons, QCA remains a legitimate and useful method as long as it is applied with caution and transparency. First, given that no statistical methods are designed for analysing the relationships between possible configurations of multiple variables and an outcome (or its absence), QCA is welcomed for its capacity to analyse such relationships. Note that this does not necessarily constitute a criticism of statistical methods, because such analysis is not statistical by nature. In addition, QCA is built on set-theory or Boolean algebra, which are two legitimate and logically sound mathematical branches; researchers criticizing QCA must show why QCA does not work under certain circumstances while its foundation remains intact. Second, all specific and technical methods involve the researcher’s subjectivity at each step of the analytical process; therefore, uncertainties arising from arbitrary decisions are common across social sciences, not a problem specific for set-theoretical methods. The best practice of dealing with such uncertainties is to explicitly incorporate the analysis of these uncertainties in the study and to make the whole research process transparent so that other members of the scientific community will be able to make their own evaluations collectively. It is in this spirit that the following analyses were conducted.

## Data and method

### Source of data

To discover causal conditions for loneliness among adults in the UK, the author analysed the data collected from the UK sample of European Social Survey Round 6 (2012). The ESS is designed to discover and explain Europe’s changing institutions, attitudes, beliefs, and behaviours across the populations in participating nations. The target population is all adults in private residence. In each of these nations, a nationally representative random sample was interviewed face-to-face. The interested reader could find further information in Jowell et al. (2007) and the ESS website (www.europeansocialsurvey.org).

At the time of writing, Round 6 is the latest available dataset that contains the needed information about loneliness and the interested causal conditions. Whilst other researchers have analysed the data collected in Round 3 (2006) for discovering risk factors for loneliness with statistical methods (Lykes and Kemmelmeier 2014; Victor and Yang 2012; Yang and Victor 2011), this study is likely to be the first to analyse the data collected in this round. The original sample size is 2286. As the empirical focus of this study is the UK, the 92 non-UK citizens were ineligible and therefore excluded from this study, thereby reducing the sample size to 2193. Furthermore, missing values are not allowed for using QCA. As the analysis is not statistical, no attempt was made to replace them with multiple imputation methods. Consequently, thirty-one cases with at least one missing value for any of the five selected conditions (see below) were removed as well, making the final sample size for the subsequent analyses 2162.

### Measures

The outcome of analysis is the participant’s sense of loneliness in ESS Round 6, which was originally measured with the following instrument: ‘How much of the time during the past week you felt lonely?’, and the participant could choose one from the following four options: 1 = ‘None or almost none of the time’, 2 = ‘Some of the time’, 3 = ‘Most of the time’ and 4 = ‘All or almost all of the time’. Following the recommendation by Schneider and Wagemann (2012: 15) that ‘one should use *fs*QCA whenever possible’, the author has calibrated the four values into the following fuzzy membership scores of the set ‘lonely adults’: ‘None or almost none of the time’ = 0.1, ‘Some of the time’ = 0.3, ‘Most of the time’ = 0.7, and ‘All or almost all of the time’ = 0.9.

There are no hard and fast rules for determining the number of causal conditions to be included in a particular set-theoretic analysis, although the general guidance is to keep it ‘at a moderate level’ in order to avoid ‘severe problems of limited diversity’ (Schneider and Wagemann 2012: 276–277). ‘Limited diversity’ and ‘logical remainder’ describe the same problem for QCA, that is, there is insufficient data in a number of rows of the truth table for the analyst to assess the relationship between the configurations and the outcome. Obviously, it is less a problem for a large dataset such as the ESS. In principle, the number of conditions to be included in a large-N study should be determined by striking a balance between the number of possible configurations and their importance in explaining the outcome. On the one hand, the relevance of a condition derived from existing studies is an important criterion for it to be included. In the literature of loneliness, a large number of theoretically important conditions (or risk factors) have been identified, including gender, age, marital status, living arrangement, health conditions and social relations (Anderson 1998; Cacioppo et al. 2010; Prieto-Flores et al. 2011; Victor et al. 2000). On the other hand, as the number of possible configurations (2^*k*^) increases exponentially with the number of conditions (*k*), to include all important conditions will make the analysis overwhelmingly complex, which is why none of the existing large-N QCA studies contains more than five conditions. Therefore, here the number of conditions is limited to five (or 32 possible configurations), making the sample size (2162) more than 67 times of the number of conditions, which is far more than the minimum of four times suggested by Marx and Duşa (2011).

The rest of this section explains the rationale for selecting and calibrating the conditions in this study. The first is age. Loneliness is widely perceived to be a problem for older people (Arnold-Cathalifaud et al. 2008; Sauer 2006; Tan et al. 2004). Although some researchers have pointed out that loneliness is a serious mental health problem for adolescents as well (Sahin 2012; Storch and Masia-Warner 2004), adolescents were not eligible for participating in the ESS. Note that the question here is whether membership of the set ‘older people’ is a necessary or sufficient condition for feeling lonely, not the statistical association between age and loneliness, as analysed in other studies (Yang and Victor 2011). At what age the word ‘old’ or ‘older’ would be deemed appropriate is highly controversial. Furthermore, as shown in a survey commissioned by the UK’s Department of Work and Pensions (Adams 2013), young people tend to give an earlier age for being old while older people a more advanced one. To be as consistent with public perception as possible, the calibration of age draws on the result from the UK sample of Round 4 of ESS (2008), in which respondents were asked ‘At what age do you think people generally start being described as old?’ The mean is 58.16; therefore, it is sensible to treat those aged below 58 as more out than in the set of ‘old adults.’ In addition, as those aged 80 and above are usually categorized as ‘oldest old’, a score of 0.9 should be appropriate. The calibrated membership scores are presented in Table 1.

**Table 1:** Calibrations and descriptive statistics

Condition	Original values	Calibrated membership	%
Lonely	All or almost all time	0.9	2.0
	Most of the time	0.7	5.0
	Sometimes	0.3	22.8
	Never or almost never	0.1	70.2
Age	≤29	0.1	14.2
	30–39	0.2	13.9
	40–49	0.3	16.3
	50–57	0.4	13.6
	58–64	0.6	11.9
	65–70	0.7	8.5
	71–79	0.8	13.0
	≥80	0.9	8.6
Gender	Female	1	57.6
	Male	0	42.4
Living with spouse/partner	Yes	1	53.6
	No	0	46.4
Self-reported health	Very good	1	27.5
	Good	0.8	41.0
	Fair	0.6	22.2
	Bad	0.3	7.9
	Very bad	0.1	1.4
Frequency of socially meeting with friends, relatives or colleagues	Everyday	1	12.8
	Several times/week	0.9	29.4
	Once/week	0.8	22.4
	Several times/month	0.6	13.5
	Once/month	0.4	10.1
	Less than once/month	0.2	9.2
	Never	0	2.6

Besides age, gender and marital status are the other two demographic variables widely included in existing studies on loneliness (de Jong Gierveld 1987; Hawkley and Cacioppo 2010; Victor and Yang 2012). As females are found to be more likely to be lonely than males, ‘gender’ is calibrated into a condition indicating whether a respondent belongs to the crisp set of ‘females’ (1); in the ESS, the only alternative option is ‘male’ (0). Of different types of ‘marital status’, widowhood has been found to be strongly associated with loneliness; however, ‘marital status’ is a multi-value condition, and to study each value as a necessary or sufficient condition would make the analysis over-stretched. In addition, whether an adult lives with their spouse (or partner) logically implies whether the respondent is married or in civil partnership, which is more important than marital status in affecting loneliness. It is therefore sufficient to use ‘living with spouse or partner’ as a much simpler condition. Moreover, in the spirit of QCA, these demographic conditions should not be treated as ‘independent’ or ‘control’ variables, as they are in statistical models; rather, they are analytically on equal footing as other conditions to be specified below.

Physical health is another factor widely identified to be responsible for loneliness. The condition to be included here is ‘self-reported health’, which has five original values: ‘Very good’ (1), ‘Good’ (2), ‘Fair’ (3), ‘Bad’ (4), and ‘Very bad’ (5). As commonly recommended to the application of *fs*QCA, the threshold or ‘crossover’ value of 0.5 is avoided due to its maximum ambiguity. The interest here is to discover whether a respondent belongs to the set of ‘perceiving oneself as physically healthy’, and it is therefore sensible to treat ‘Fair’ as ‘just in’ with a membership score of 0.6. Accordingly, ‘Good’ has a score of 0.8, and ‘Very good’ has full membership of 1; those in ‘Bad’ health are clearly out of this set (not healthy), therefore are assigned a membership score of 0.3; similarly, those in ‘Very bad’ health have the membership of 0.1.

Finally, a condition that describes the respondent’s social relations must be included. Round 6 of ESS contains three such conditions: ‘How often socially meet with friends, relatives or colleagues’, ‘How many people with whom you can discuss intimate and personal matters’, and ‘Take part in social activities compared to others of same age’. Statistically, they are expected to be strongly correlated and therefore become components of a composite index of sociality. Indeed, the author’s initial plan was to create such a composite index as one condition by summing up the scores of these three variables. It turns out, however, that the level of association among them is not sufficiently strong for such purpose (Cronbach’s alpha = 0.53). That is, these three questions do not seem to be about one latent factor and as a result, each of them must be calibrated individually. However, with four conditions already selected, adding yet three more conditions will make the analysis extremely complicated. In the end, only the first one is included as it has the face validity of measuring the respondent’s level of sociality. The second instrument will be used later as an alternative measure of sociality for testing the robustness of the results.

The frequency of social meetings, originally measured with the values of ‘How often socially meet with friends, relatives or colleagues,’ is taken as the membership of the set of ‘social persons.’ A respondent has full membership (1) if meeting others every day and (0) if never; in between are five fuzzy-set scores: 0.2 for ‘less than once a month’, 0.4 for ‘once a month’, 0.6 for ‘several times a month’, 0.8 for ‘once a week’, and 0.9 for ‘several times a week’.

Sampling weights are not used in QCA as the analysis does not aim to estimate any population parameters with sample statistics. Calibrated memberships of each of the selected conditions with respective percentage are presented in Table 1.

## Results

Schneider and Wagemann suggest that the ‘analysis of necessary conditions should be separate from and should precede the analysis of sufficient conditions’ (2012: 278). Accordingly, the results from the analysis of necessity will precede those from sufficiency analyses. All results in this section were produced with the software programme *fs*QCA (Ragin and Davey 2014).

### Necessary conditions for loneliness

The process of analysing necessity starts with each selected condition. Necessity is determined by the consistency statistic, which is measured as the percentage that the cases whose membership of the condition is equal to or greater than their membership in the outcome, and it is highly recommended that the consistency level be no lower than 0.9 (Ragin 2006). If the consistency level does not reach 0.9, another or even a third condition will be added to construct a union of two or more conditions, which is expected to have a higher level of consistency. In addition to consistency, the coverage of a condition measures the extent to which the outcome is a subset of the condition. These conditions and their corresponding consistency and coverage levels are presented in Table 2. Obviously, there are many two-condition and three-condition unions, and only the theoretically relevant ones are presented.

**Table 2:** Analysis of necessary conditions for loneliness

Condition	Symbol	Consistency	Coverage
*Single conditions*			
Old age	O	0.844	0.354
Female	F	0.599	0.199
Male	M	0.401	0.181
Not living with spouse/partner	~Lw	0.593	0.245
Not having good health	~H	0.622	0.499
Not being social	~S	0.666	0.442
*Selected unions of two conditions*			
Old age + female	O + F	0.951	0.236
Old age + not living with spouse/partner	O + ~Lw	0.960	0.258
Old age + poor health	O + ~H	0.888	0.344
Old age + not social	O + ~S	0.900	0.320
Female + not living with spouse/partner	F + ~Lw	0.824	0.210
Male + not living with spouse/partner	M + ~Lw	0.769	0.207
Not living with spouse/partner + not social	~Lw + ~S	0.925	0.278
Not living with spouse/partner + poor health	~Lw + ~H	0.845	0.279
*Selected unions of three conditions*			
Old age + female + not living with spouse/partner	O + F + ~Lw	0.991	0.217
Old age + female + poor health	O + F + ~H	0.966	0.235
Old age + female + not social	O + F + ~S	0.967	0.230
Old age + male + not living with spouse/partner	O + M + ~Lw	0.969	0.222
Old age + male + poor health	O + M + ~H	0.922	0.250
Old age + male + not social	O + M + ~S	0.933	0.243

The results clearly show that, of the five single conditions, only ‘old age’ has a consistency level close to 0.9 (0.844); all other conditions’ consistency levels are far below 0.9. Moving on to the four two-condition unions that contain ‘old age’, three have consistency levels equal to or above 0.9 (‘old age or female’, ‘old age or not living with spouse/partner’, and ‘old age or not social’); the consistency of ‘old age or poor health’ is just short of 0.9. In short, an advanced age by itself is not a necessary condition for loneliness, but its conjunction with any one of the other four conditions is. Another two-condition union with a consistency level above 0.9 is ‘Not living with spouse/partner or Not social’. The consistency levels of the rest of the two-condition unions are close but below 0.9. Finally and not surprisingly, when a third condition is added to a two-condition union, all necessary conditions have consistency levels above 0.9.

### Sufficient conditions for loneliness

In QCA, the analysis on sufficiency relies on the truth table. In this study, the truth table (Table 3) includes the following five conditions: ‘female’ (F) and ‘not living with spouse/partner’ (~Lw) are crisp sets, while ‘old age’ (O), ‘self-reported bad health’ (~H), and ‘not frequently being social with others’ (~S) are fuzzy sets. Understandably, for a large-N dataset such as the ESS, a higher frequency threshold (or the minimum number of cases for each configuration) should be used (Ragin 2008a: 78); here, the frequency threshold of 5 instead of 1 or 2, which are usually used in small-N analysis, is applied. Thus, more than 99% of the cases will be included in the subsequent analyses.

**Table 3:** Truth table for analysis of sufficiency on loneliness

No.	O	F	~Lw	~H	~S	Number of cases	Lonely	Raw consistency
1	0	0	1	1	1	6	1	0.785047
2	1	1	1	1	1	11	1	0.779591
3	0	1	1	1	1	7	1	0.75989
4	1	0	1	1	1	10	1	0.757384
5	1	1	1	0	1	27	0	0.718687
6	1	0	1	1	0	15	0	0.709388
7	1	0	1	0	1	23	0	0.708527
8	0	0	1	0	1	24	0	0.682779
9	0	1	1	1	0	20	0	0.674524
10	0	0	1	1	0	18	0	0.646563
11	1	1	1	1	0	33	0	0.641233
12	1	1	0	1	1	3	0	0.636616
13	0	1	1	0	1	66	0	0.62244
14	0	1	0	1	1	13	0	0.610608
15	0	0	0	1	1	1	0	0.601706
16	1	0	0	1	1	8	0	0.563233
17	1	0	1	0	0	100	0	0.533386
18	1	1	0	0	1	37	0	0.527089
19	0	1	0	1	0	14	0	0.512267
20	1	1	0	1	0	15	0	0.499529
21	0	0	0	1	0	9	0	0.489362
22	1	0	0	0	1	53	0	0.468846
23	1	1	1	0	0	201	0	0.467887
24	0	1	0	0	1	101	0	0.452512
25	1	0	0	1	0	19	0	0.43272
26	0	0	0	0	1	85	0	0.424329
27	0	1	1	0	0	256	0	0.404535
28	0	0	1	0	0	187	0	0.374036
29	1	1	0	0	0	184	0	0.340562
30	1	0	0	0	0	170	0	0.298513
31	0	1	0	0	0	257	0	0.296468
32	0	0	0	0	0	189	0	0.284069

It is usually required that the raw consistency level be no lower than 0.75 (Ragin 2008a: 78; Schneider and Wagemann 2012: 279), which is more acceptable when the sample size is as large as that of the one used here. The first four rows of Table 3 show the configurations whose consistency level is above 0.75. Note, however, that other than configurations 12 and 15, which has three cases and one case, respectively, these four configurations are among the least popular ones, an issue to be discussed in the next section. In contrast, the most popular configurations are 27 (256 cases) and 31 (257 cases), which together count as 15.5% of the sample size, who are old, female, not healthy, not frequently social with others, either living or not living with spouse or partner.

The next step is to derive a solution that is logically the most efficient based on the four configurations with consistency levels above 0.75. For this study, finding the solution is straightforward: the configurations have the value of 1 for ‘not living spouse/partner’, ‘not healthy’, and ‘not being frequently social with others’; and for the other two conditions, ‘being old’ and ‘being female’, two rows have 1 and the other two 0. Thus, these latter two conditions are logically redundant and the intersection of the other three is sufficient. The computer programme *fs*QCA routinely generates three different logical solutions, complex, parsimonious, and intermediate, depending on the assumptions of counterfactuals used (for further explanations, see Schneider and Wagemann 2012). For this study, all three solutions are the same, which confirms the above solution based on intuition: ~Lw * ~H * ~S, with the intermediate solution having the first three conditions present and the other two either present or absent. This solution means that, for the adult citizens of the UK, ‘not living with spouse/partner and not being healthy and not being social’ is a sufficient condition for feeling frequently lonely. The raw, unique and solution coverages are all equal in this case (0.239), and the solution consistency and term consistency both are equal to 0.720, which is lower than the usually expected 0.75 or 0.8 but satisfactory given the large number of cases.

### Test of robustness with two different conditions

As the solutions to truth tables are sensitive to the selection of conditions, the calibration of their values, and the thresholds, it is advisable to test the robustness of the solutions with different choices. The limited space will only allow one further analysis with two different conditions. In this analysis, the outcome, age, gender, and ‘whether living with spouse/partner’ remain the dataset. The self-reported health used previously is replaced with another variable that indicates whether the respondent was ‘hampered in daily activities by illness/disability/infirmity/mental problem.’ The original values of this variable are now calibrated as follows: 0.9 for ‘Yes a lot’, 0.7 for ‘Yes to some extent’, and 0 for ‘No’. The ‘frequency of being social with others’ used above is replaced with the variable ‘How many people with whom you can discuss intimate and personal matters.’ In order to make them comparable with those used for the variable ‘self-reported health’ used in the previous analysis, the values of this variable are calibrated as follows: 0 for ‘None’, 0.2 for 1, 0.3 for 2, 0.4 for 3, 0.6 for ‘4–6’, 0.8 for ‘7–9’, and 0.9 for ‘10+’; that is, this set could be labelled as ‘having four or more confidantes.’ The truth table now consists of the following conditions: ‘old’, ‘female’, ‘not living with spouse/partner’, ‘having illness hampering daily life’, and ‘not having at least four confidantes.’ No results are presented here, however, as the highest consistency level is only 0.6742. Even for a large-N dataset, most researchers using *fs*QCA would not accept such low level of consistency. Although these two conditions have generated different results, they do not help discover other sufficient conditions for loneliness.

### Analysis of sufficiency on the negation of the outcome

As pointed out previously, an advantage of QCA is analysing the presence and the absence of the outcome separately (or asymmetrical causal relationship): that X is a cause of Y does not necessarily mean that not X is the cause of not Y. For this study, the analysis of the negation of the outcome could be particularly useful after it is discovered that the percentage of cases with consistency levels above the required 0.75 is very small, which is likely due to the small percentage of people who were lonely—only 2% were lonely all or almost all the time and 5% lonely most of the time. For these two categories of the outcome, only 34 of 2162 cases, or 1.57%, belong to the configurations with a consistency level above 0.75; the other 98.43% of the cases are treated as logical remainders (configurations without cases for the outcome). Given the large size of this sample, it seems sensible to lower the consistency threshold. However, as Table 4 shows, even when the threshold is lowered to 60%, only 12.8% of the cases are in configurations with consistency levels above the threshold. These strongly suggest that the heavily unbalanced distribution of the cases across different memberships of loneliness must be taken into account when examining the results.

**Table 4:** Consistency threshold and percentage of cases in configurations with consistency levels above the threshold

Threshold of consistency (%)	% cases in configurations
75	1.57
70	4.58
65	6.61
60	12.8

If the above reasoning is correct, then the consistency levels for the negation of the previous outcome, i.e., not lonely, are expected to rise. The truth table (Table 5) confirms that this is indeed the case, in which ‘not lonely’ is the outcome, and five theoretically sensible conditions are included: male, not old, living with spouse/partner, healthy, and frequently being social with others. This time, even the lowest consistency is close to 0.94, well above the required 0.75 or 0.8. As configuration No. 1 has only one case, it was not included in the following analysis for solutions.

**Table 5:** Truth table for analysis of sufficiency on not feeling lonely

No.	O	M	Lw	H	S	Number of cases	Raw consistency
1	1	1	1	0	0	1	0.99431
2	0	1	1	0	1	19	0.994192
3	0	1	1	0	0	8	0.993481
4	0	1	1	1	1	170	0.993292
5	1	0	1	0	1	14	0.992149
6	1	1	1	1	1	189	0.991713
7	0	0	1	0	0	3	0.991678
8	1	1	1	0	1	9	0.991041
9	0	0	1	1	0	37	0.98839
10	1	1	1	1	0	85	0.986966
11	0	0	1	0	1	15	0.98683
12	0	0	1	1	1	184	0.98525
13	1	0	1	0	0	13	0.980595
14	0	1	1	1	0	53	0.978193
15	1	0	1	1	1	257	0.977666
16	1	1	0	1	0	24	0.97281
17	1	1	0	0	0	6	0.971963
18	1	0	1	1	0	101	0.971446
19	0	0	0	1	0	27	0.966203
20	1	1	0	0	1	18	0.962132
21	1	0	0	0	0	7	0.961801
22	0	1	0	1	0	23	0.96124
23	1	1	0	1	1	187	0.955995
24	0	1	0	0	1	15	0.95231
25	1	0	0	0	1	20	0.951949
26	0	1	0	1	1	100	0.949316
27	0	0	0	0	0	11	0.946939
28	1	0	0	1	0	66	0.946765
29	0	1	0	0	0	10	0.943038
30	1	0	0	1	1	256	0.942911
31	0	0	0	1	1	201	0.938838
32	0	0	0	0	1	33	0.938312

Table 6 presents the intermediate solution to Table 5 by assuming the presence of ‘living with spouse/partner’, ‘healthy’, and ‘being frequently social with others’, with ‘being old’ and ‘male’ are either present or absent. Five separate sufficient conditions are identified, three of which have consistency levels above 0.9: not old, healthy, and being frequently social with others; given that the consistency level of every configuration is above 0.9, these three solutions should be accepted. The other two solutions—being female and not living with spouse/partner—have lower consistency levels, do not make sense substantively, and therefore are not taken as solutions.

**Table 6:** Intermediate solution for Table 5

Frequency cutoff	3
Consistency cutoff	0.938
Assumptions	S, H, and Lw are present

	Row coverage	Unique coverage	Consistency
~O	0.531	0.004	0.938
F	0.570	0.017	0.801
~Lw	0.434	0.005	0.755
S	0.801	0.012	0.910
H	0.852	0.030	0.905
Solution coverage	0.984		
Solution consistency	0.822		

## Discussion

This paper set out to discover the necessary and sufficient causal conditions for loneliness among adults in the UK by analysing the UK sample of the ESS (Round 6) with QCA, a set-theoretic method that has never been used before on this problem. The intention was to advance our understanding of loneliness by making use of some unique features of QCA, including the identification of necessary and sufficient conditions separately, the discovery of necessary and sufficient configurations as opposed to each predictor’s unique contribution to the explanation of the response variable, and the revelation of asymmetrical relationships between the configurations and the outcome. As demonstrated in the previous section, these analyses could be very sensitive to the selection of conditions, the choice of thresholds, and the distribution of cases. This section discusses the extent to which the planned objectives have been achieved.

Findings from this study complement existing statistical analyses of the relationship between age and loneliness, either linear or non-linear (Yang and Victor 2011; Victor and Yang 2012). While statistical methods analyse all quantitative values of age, QCA focuses on the qualitative boundaries of the set ‘older people’, even though such boundaries are quantitative. This study thus intends to find out whether it is the *identity* of being a member of ‘older people’, *not merely any numeric value of age* that induces loneliness. But perhaps the greatest contrast between findings from this study and those from others is that an old age (or more precisely, a higher than 0.5 membership of the set ‘aged 58+’) per se is not a necessary condition; it is only when old age is united with one of the other four conditions that it becomes necessary. Being ‘older’ is not sufficient for frequent loneliness either, but ‘not being older’ appears to be sufficient for not being lonely. Given the deterministic nature of QCA, this is not surprising: for example, of all the respondents aged 60+ in Round 6 of the ESS, 69.8% were never or almost never lonely; similarly, it is possible albeit rare that some people are female, not living with spouse or partner, not healthy, and not frequently social with others but are not lonely. Conversely, those who are lonely are not all female, old, not living with spouse or partner, poor in health, and not frequently social with others. This study confirms that such observations are valid even after the fuzziness of each condition’s values has been taken into account.

To focus on configurations rather than a collection of individual variables is another advantage of QCA over linear statistical models. Studying the configurations of conditions could answer research questions that linear statistical models cannot. For the study of loneliness, the question ‘Who are lonely?’ is categorically different from ‘Which risk factors are statistically significant?’—that gender, age, marital status, health and social relations each turns out to be statistically significant does not lead to a meaningful answer to the question ‘Are old and widowed females in poor health and not being frequently social with others necessarily lonely?’ and ‘Are those in the opposite configuration necessarily not lonely?’. Using QCA, this study has found that the scores of these unions are equal to or higher than the fuzzy membership of loneliness. In other words, an old age matters only when it is in conjunction with one of these other conditions for loneliness.

Although age appears in these two-condition necessary unions for being lonely, neither age nor gender appear in the sole solution for sufficiency (not living with spouse/partner and not healthy and not being frequently social with others), which provides new evidence for the spurious effects of these two widely researched demographic risk factors. If loneliness is a mental suffering due to the absence of desired social relations, any observed association between age and gender must be spurious as such association must have come from an unspecified and undesirable situation of social relations. Even when old age and being female are combined, it is only a necessary but not sufficient condition. Moreover, the solution ‘~Lw * ~H * ~S’ also indicates that it is actually very unlikely for adults in the UK to feel lonely because one must satisfy these three conditions simultaneously, which is not surprising given that the percentage of the frequently lonely among all adults in the UK is lower than 10%. In other words, this study offers a new explanation for why the percentage of lonely adults in the UK is so low.

Greater caution has been applied in conducting this analysis due to the criticisms of the set-theoretic method in general and QCA in particular. However, some issues and limitations are inherent to this approach. The first issue relates to the relationship between the sample size and the number of conditions to be included in the analysis, which has not been properly resolved among methodologists of QCA. A large sample size will make it less likely to have many logical remainders, but this is only true if the number of conditions included remains small, which could be theoretically constraining. Although the ESS sample used in this study enjoys a large size, some important conditions for understanding loneliness, such as immigration, ethnicity and employment, could not enter the analysis.

Another issue arising from this study is the percentage of cases in the configurations whose consistency levels are above the predetermined threshold. In this study, it is very low when the outcome is being lonely. When the outcome switches to its opposite, every configuration has a very high consistency level. It is clear that such uncertainty comes from the distribution of cases across the values of the outcome. Researchers using QCA are therefore urged to pay careful attention to the effect of such distribution on the results.

## Conclusions

Using QCA, this study has produced some causal analyses of loneliness that are different from those produced with statistical methods. Here loneliness is measured with fuzzy set memberships, rather than composite scores or self-reported frequencies. The causes of loneliness are studied as configurations rather than individual risk factors. Whilst gender is widely identified as a statistically significant risk factor in existing studies, it is neither necessary nor sufficient for loneliness. Old age by itself is not a necessary condition either and only becomes necessary when it is combined with another condition. The only sufficient causal condition for loneliness is not living with spouse/partner and not being healthy and not being social with others at the same time. In great contrast to symmetrical associations between loneliness and its risk factors that are revealed in statistical models, an asymmetrical relationship is found: the negation of some conditions is a sufficient condition for not being lonely, and the sufficient condition for being lonely is different from the sufficient condition for *not* being lonely. Readers who find these findings too deterministic need to understand that this is the very nature of QCA and the results are better understood in connection to relevant statistics.

This study has produced some methodological insights as well. To use QCA appropriately, particularly on a large-N dataset, the analyst must carry out a series of separate analyses that vary across different situations, including the conditions included, different calibrations, different thresholds, different distribution of the cases across the values of the outcome, and both the presence and the absence of the outcome. Without detailed knowledge about the cases, as researchers usually have when the sample size is small, carrying out these separate analyses and carefully developing a coherent account from them is the only way to ensure the high quality of such analysis.

Identifying combinations of attributes and their relations with the interested outcome, loneliness in this study, is effectively a process of classification as well, because these combinations describe the shared attributes of a group of cases. The discovery of such configurations can help policy-makers and practitioners identify the targets of interventions. This kind of targeting could be more effective than those derived from statistical models; for example, while statistical analyses may show that the widowed, those with physically limiting illness, and having no intimate relationship are the most vulnerable to loneliness, what they effectively claim is that the presence of any one of these attributes will have a significant effect on the likelihood of loneliness, but in reality to feel lonely actually requires the combination of two or three conditions at the same time.
